# The Orthoptera of Castro Verde Special Protection Area (Southern Portugal): new data and conservation value

**DOI:** 10.3897/zookeys.691.14842

**Published:** 2017-08-17

**Authors:** Sílvia Pina, Sasha Vasconcelos, Luís Reino, Joana Santana, Pedro Beja, Juan S. Sánchez-Oliver, Inês Catry, Francisco Moreira, Sónia Ferreira

**Affiliations:** 1 CIBIO/InBIO-UP, Centro de Investigação em Biodiversidade e Recursos Genéticos, Universidade do Porto. Campus Agrário de Vairão, Rua Padre Armando Quintas, 4485–601, Vairão, Portugal; 2 CEABN/InBIO, Centro de Ecologia Aplicada “Professor Baeta Neves”, Instituto Superior de Agronomia, Universidade de Lisboa, Tapada da Ajuda, 1349-017 Lisboa, Portugal; 3 REN Biodiversity Chair, CIBIO/InBIO-UP, Centro de Investigação em Biodiversidade e Recursos Genéticos, Universidade do Porto, Campus Agrário de Vairão, Rua Padre Armando Quintas, 4485–601 Vairão, Portugal

**Keywords:** Biodiversity, open-habitats, species inventory, distribution extension

## Abstract

With the increasing awareness of the need for Orthoptera conservation, greater efforts must be gathered to implement specific monitoring schemes. Despite recent surveys, little is known about Portuguese Orthoptera populations. This study was performed in 2014 and 2015 mainly in Castro Verde Special Protection Area (SPA), southern Portugal, and is the first Orthoptera inventory conducted in the area. A total of 35 Orthoptera species was recorded, with two new species reported for Portugal. We provide species’ habitat occurrences within the protected area and use information on the conservation status and the Iberian distribution of each documented species to discuss the importance of Castro Verde SPA for Orthoptera conservation. The data presented here sheds new light on Castro Verde SPA biodiversity and emphasizes the inclusion of this area in the conservation of Orthoptera diversity, particularly in the protection of threatened endemic species.

## Introduction

The results of the recent Red List assessment of Europe’s grasshoppers, crickets and bush-crickets indicate that the highest species diversity and the greatest concentration of threatened species are found in the Mediterranean region. Moreover, the highest number of Data Deficient species are found in the Mediterranean region and many are also found in the Iberian Peninsula along with endemic species (Hochkirch et al. 2016). The climate and ecological conditions in the Iberian Peninsula promote the presence of an abundant and varied orthopterofauna (Llucià-Pomares 2002). To preserve this biodiversity, it is essential to increase our knowledge on species distributions. Therefore, taxonomic studies and faunistic inventories at a local and regional level should be intensified.

On the Iberian Peninsula, the Orthoptera fauna has been more comprehensively studied in Spain, while there is still a lack of knowledge regarding the Portuguese Orthoptera (Schmidt et al. 2009). To find the first studies on the Orthoptera fauna of Portugal we have to consult the literature of the last quarter of the XIX century. Bolívar (1876) published the first study about this group for Portugal and Spain, although some species had been previously referred to by Baptista (1789), Vandelli (1797), Charpentier (1841–1845) and Fischer (1853). More recently, several Portuguese and foreign researchers have contributed to improve the knowledge of the Portuguese Orthoptera fauna, e.g. Lock (1999); Miranda-Arabolaza and Barranco (2005); Ferreira et al. (2006, 2007, 2008, 2009); Ferreira (2007, 2009); Ferreira and Grosso-Silva (2008a, b, c); Schmidt et al. (2009); Lemos et al. (2016); Monteiro et al. (2016). However, these studies are scattered over time, distinct in focus, and generally lack comprehensive inventories. There are thus numerous unexplored Portuguese regions regarding the knowledge of the orthopterofauna. As a consequence, ten species have been assessed as Data Deficient in continental Portugal (Hochkirch et al. 2016). Moreover, in recent studies, several species were reported for the first time for the country, e. g. Lemos et al. (2016), Monteiro et al. (2016). These evidences suggest that there is still much to learn about this group, and that further in-depth research on Orthoptera of Portugal is necessary.

The Castro Verde Special Protection Area (SPA, PTZPE0046, Natura 2000 network) consists mainly of extensive cereal-steppes, however in recent years the afforested area has increased. The area houses the most significant diversity and abundance of steppe birds in Portugal and, therefore, was designated for steppe bird conservation under the European Union Birds Directive (79/409/EEC). Several bird species listed in Annex I of the Birds Directive are regularly found there, including the lesser kestrel (*Falco naumanni*), the great bustard (*Otis tarda*) and the little bustard (*Tetrax tetrax*). While the biological and conservation importance of birds in Castro Verde SPA has long been recognized, little is known about its value for other groups, namely for arthropods. Many orthopterans, especially grasshoppers, are highly dependent on grassland habitats and are major primary herbivores (Ingrisch and Köhler 1998, Köhler et al. 1987). Furthermore, orthopterans are important components in grassland ecosystems, often accounting for the largest biomass of arthropods (Little et al. 2013). On the other hand, they are an important food resource for a range of species, some of which are of conservation concern. For example, *O. tarda*, *T. tetrax* and *F. naumanni* consume orthopterans during the breeding and pre-migration periods (Rocha et al. 2005, Jiguet 2002, Catry et al. 2014, Bounas and Sotiropoulosa 2017). Despite its importance, to date no comprehensive inventory on Orthoptera fauna has been performed in Castro Verde SPA. The only available information comes from the work of Schmidt et al. (2009), who conducted surveys between 1992 and 2000 in several regions of Portugal, and recorded six species from Castro Verde.

With this study, we aim to increase the knowledge of the Orthoptera fauna in Portugal, presenting a list of species and the respective habitats of occurrence in a fragmented and human-altered landscape. We gather information on the conservation status and distribution of each documented species, and discuss the importance of the Castro Verde SPA for the conservation of Iberian Orthoptera diversity.

## Methods

**Figure 1.**
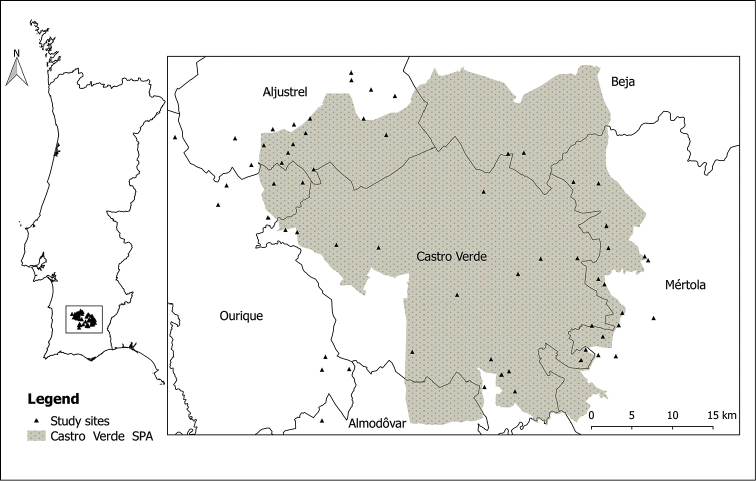
Location of the study area in southern Portugal showing the sites sampled (triangles), the Special Protection Area of Castro Verde, and the six municipalities encompassed.

The data presented in this paper are a result of fieldwork performed in a farmland landscape mostly included in the Castro Verde SPA, southern Portugal (Figure 1). The climate is Mediterranean, with hot summers (averaging 24°C [16–32°C] in July), mild winters (9°C [5–14°C] in January) and >75% of annual rainfall (500–600 mm) concentrated in October–March. The area is the most representative steppe area in Portugal, with 85.345 hectares of which around 60.000 hectares are pseudo-steppe (EC 2016). The landscape is flat or gently undulating (100–300 m a.s.l.) and dominated by an agricultural mosaic of cereal, pastures and grazed fallow land (Ribeiro et al. 2016). Tree cover is characterized by plantations of eucalyptus (*Eucalyptus* sp.), oaks (*Quercus rotundifolia* and *Q. suber*) and umbrella pines (*Pinus pinea*). Pine and oak plantations often have a grassy understorey grazed by livestock. The area occupied by forest plantations increased since the 1990’s, mostly in the periphery of the Castro Verde SPA, due to Common Agricultural Policy (CAP) subsidies for farmland afforestation (Reino et al. 2010). Nevertheless, specifically within the protected area afforestation is currently prohibited.

To study species diversity and habitat specificity, orthopterans were visually recorded along transects placed in the different habitats. This method was chosen because it has been widely used to sample Orthopteran (Gardiner et al. 2005), and because it provided a simple and standardised approach to sample a large number of sites with different vegetation structure within a relatively short period. Sampling was performed in 61 sites, each composed of one forest plantation and one adjacent fallow field (Table 1). The forest plantations consisted of one of the following types: pine, oak, eucalyptus and mixed pine-oak. Sampling was performed in 32 sampling sites in 2014 and the remaining 29 sites in 2015, in three periods each year (2014: 15 April–15 May, 15 May–4 June, 13 June–1 July; 2015: 16 April–17 May, 18 May–1 June, 15 June–23 June). Surveys were carried out under similar meteorological conditions (sunny weather, with temperature > 17º C and without strong winds), during the periods of highest activity of most orthopterans (9:00 am to 6:00 pm). Linear transects 50m long were placed in each of the forest plantation, adjacent fallow and the edge between both, and within each site we sampled 1 transect at the habitat edge, 3 transects in fallows and 1-3 transects in plantations, depending on the forest patch size. Orthopterans were recorded along the 50-m transects, within 50cm on either side of the observer, and collected by direct capture a representative sample of adults that appeared to be morphologically distinct. To complement the species inventory, within each sampling site we further collected individuals recorded outside the standardised 50-m transects, which appeared to be morphologically distinct. All individuals collected were later identified to species level in the laboratory, based on: Llucià-Pomares (2002), Defaut (2005a) and Defaut and Aulin (2008) for *Aiolopus* spp.; Barat (2012) for Bradyporinae; Defaut (2004) and García et al. (2005) for *Dociostaurus* spp.; Ragge and Reynolds (1984) for *Euchorthippus* spp.; Llorente and Pinedo (1990) for *Odontura* spp.; Cordero et al. (2009) for *Oecanthus* spp.; Defaut (2006) for *Oedipoda* spp.; Clemente et al. (1990) for *Omocestus* spp.; Massa (2012) and Llorente and Presa (1997) for Pamphagidae; and Husemann et al. (2013) and Defaut (2005b) for *Sphingonothus* spp. Scientific nomenclature follows Cigliano et al. (2016) and vouchers of each species are deposited in CIBIO’s collection.

**Table 1.** Information on the sampling sites prospected in this study: site code, municipality, locality, WGS 84 coordinates, elevation and plantation type. Each coordinate corresponds to the centre of the edge between fallow and plantation.

Sampling site	Municipality	Locality	Latitude / Longitude	Elevation (m a.s.l.)	Plantation type
A29	Aljustrel	Aljustrel	37°52.11'N, -8°7.152'W	206	Oak
A33	Aljustrel	Aljustrel	37°52.992'N, -8°8.484'W	140	Pine
A57	Aljustrel	Aljustrel	37°49.818'N, -8°11.49'W	214	Eucalyptus
A63	Aljustrel	Messejana	37°49.23'N, -8°12.336'W		217	Eucalyptus
A64	Aljustrel	Messejana	37°48.276'N, -8°13.086'W	213	Oak
A68	Aljustrel	Aljustrel	37°50.574'N, -8°7.65'W	200	Oak
A74	Aljustrel	Aljustrel	37°51.774'N, -8°5.556'W		169	Eucalyptus
A90	Aljustrel	Messejana	37°50.268'N, -8°12.27'W	229	Eucalyptus
B1	Aljustrel	Aljustrel	37°50.58'N, -8°11.196'W	223	Eucalyptus
B4	Aljustrel	Messejana	37°48.804'N, -8°12.666'W	241	Eucalyptus
P27	Aljustrel	Messejana	37°47.922'N, -8°10.956'W	198	Eucalyptus
P29	Aljustrel	Messejana	37°50.016'N, -8°13.692'W	220	Eucalyptus
P30	Aljustrel	Messejana	37°49.176'N, -8°14.256'W		197	Mixed pine-oak
P31	Aljustrel	Messejana	37°48.156'N, -8°15.09'W	173	Mixed pine-oak
P36	Aljustrel	Aljustrel	37°49.722'N, -8°6.138'W	178	Eucalyptus
P39	Aljustrel	Aljustrel	37°52.614'N, -8°8.466'W	150	Oak
P42	Aljustrel	Messejana	37°49.536'N, -8°16.176'W	169	Eucalyptus
A152	Almodôvar	Aldeia dos Fernandes	37°34.71'N, -8°10.398'W	258	Oak
P10	Beja	Albernoa	37°48.804'N, -7°57.006'W	169	Oak
A85	Castro Verde	Santa Bárbara dos Padrões	37°37.11'N, -7°58.482'W	238	Oak
A98	Castro Verde	São Marcos da Ataboeira	37°37.89'N, -7°53.214'W	202	Pine
B5	Castro Verde	Casével	37°44.748'N, -8°12.846'W	186	Eucalyptus
B6	Castro Verde	São Marcos da Ataboeira	37°43.254'N, -7°53.43'W	216	Eucalyptus
B14	Castro Verde	Castro Verde	37°41.322'N, -8°1.434'W	180	Eucalyptus
P1	Castro Verde	São Marcos da Ataboeira	37°43.224'N, -7°55.878'W	167	Pine
P3	Castro Verde	São Marcos da Ataboeira	37°42.414'N, -7°57.396'W	187	Oak
P8	Castro Verde	Entradas	37°46.746'N, -7°59.658'W	184	Oak
P9	Castro Verde	Entradas	37°48.75'N, -7°58.056'W		176	Eucalyptus
P16	Castro Verde	Santa Bárbara dos Padrões	37°37.932'N, -7°59.166'W	237	Eucalyptus
P17	Castro Verde	Santa Bárbara dos Padrões	37°36.48'N, -7°59.604'W	255	Pine
P18	Castro Verde	Santa Bárbara dos Padrões	37°37.29'N, -7°57.996'W	232	Oak
P19	Castro Verde	Santa Bárbara dos Padrões	37°36.258'N, -7°57.594'W	253	Oak
P20	Castro Verde	São Marcos da Ataboeira	37°38.436'N, -7°52.902'W	194	Mixed pine-oak
P22	Castro Verde	São Marcos da Ataboeira	37°39.708'N, -7°52.488'W	192	Pine
P24	Castro Verde	Castro Verde	37°43.818'N, -8°6.648'W	220	Eucalyptus
P25	Castro Verde	Castro Verde	37°43.962'N, -8°9.432'W	227	Oak
P28	Castro Verde	Castro Verde	37°38.322'N, -8°4.398'W	233	Oak
P33	Castro Verde	Casével	37°44.634'N, -8°12.06'W	190	Oak
A76	Mértola	São João dos Caldeireiros	37°38.148'N, -7°52.062'W	197	Pine
A78	Mértola	São João dos Caldeireiros	37°38.094'N, -7°50.886'W	183	Pine
A79	Mértola	São João dos Caldeireiros	37°40.116'N, -7°48.366'W	142	Pine
A99	Mértola	Alcaria Ruiva	37°39.732'N, -7°50.694'W	170	Pine
B7	Mértola	Alcaria Ruiva	37°43.14'N, -7°48.732'W	168	Eucalyptus
B8-1	Mértola	Alcaria Ruiva	37°43.35'N, -7°48.954'W	184	Eucalyptus
P5	Mértola	Alcaria Ruiva	37°43.794'N, -7°51.384'W	196	Eucalyptus
P6	Mértola	Alcaria Ruiva	37°44.958'N, -7°51.522'W	169	Pine
P11	Mértola	Alcaria Ruiva	37°47.262'N, -7°53.688'W	152	Oak
P12	Mértola	Alcaria Ruiva	37°47.184'N, -7°52.05'W	148	Oak
P13	Mértola	Alcaria Ruiva	37°41.868'N, -7°51.654'W	172	Pine
P15	Mértola	Alcaria Ruiva	37°40.386'N, -7°50.454'W	162	Pine
P23	Mértola	São João dos Caldeireiros	37°39.138'N, -7°51.756'W	173	Pine
P52	Mértola	Alcaria Ruiva	37°42.162'N, -7°52.062'W	175	Pine
B3	Ourique	Panóias	37°46.044'N, -8°17.31'W	153	Pine
B13-2	Ourique	Conceição	37°47.232'N, -8°11.688'W	194	Mixed pine-oak
P32	Ourique	Conceição	37°47.172'N, -8°13.614'W	202	Oak
P46	Ourique	Conceição	37°47.076'N, -8°16.746'W	161	Pine
P47	Ourique	Ourique	37°37.374'N, -8°10.398'W	257	Oak
P48	Ourique	Ourique	37°38.058'N, -8°10.17'W	249	Oak
P49	Ourique	Ourique	37°37.41'N, -8°8.616'W	249	Mixed pine-oak
P50	Ourique	Panóias	37°49.602'N, -8°20.154'W	118	Oak
P51	Ourique	Conceição	37°45.384'N, -8°13.986'W		186	Pine

For each species the following information is provided: sampling site, collection date, habitat occurrence and number of males (M) and females (F) recorded. Species are coded according to their occurrence in the studied habitats, as follows: FAL – fallow; EDG – edge; OAK - oak plantation; PIN – pine plantation; EUC – eucalyptus plantation; MIX – mixed pine-oak plantation. The current distribution of each documented species in the Iberian Peninsula is also given, along with a remark when our findings indicate an extension of the known species’ distribution range. Information about the conservation status of each species is also provided (see Suppl. material 1). Distribution data and conservation status are based on Hochkirch et al. (2016).

To evaluate the conservation value of the different habitat types, we calculated the Grasshopper Conservation Index (GCI) and the standardised Grasshopper Conservation Index (GCIn) proposed by Matenaar et al. (2015) (see Suppl. material 1). As proposed by Matenaar et al. (2015) the index was calculated for each recorded species using three parameters: “endemism”, “dispersal capacity” and “rarity”. “Endemism” was scored as: “1” - species with a large distribution range not confined to the Mediterranean Basin; “2”- species when endemic to the Mediterranean basin and “3” species endemic to the Iberian Peninsula. For “dispersal capacity”, scoring was made according to the following criteria: “1” - fully capable of flight; “2” - wing-dimorphic and “3” - flightless. Rarity was scored based upon the occurrence of a species in the sampling sites: rare (“3”) when it occurred at ≤5 sites, intermediate (“2”) at ≤10 sites and common (“1”) at >10 sites.

## Results

**Figure 2.**
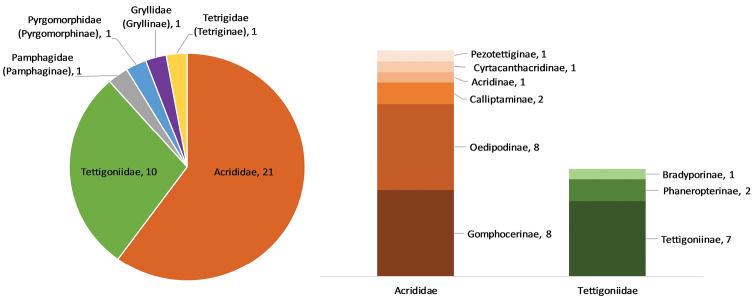
Total number of Orthoptera species per family and per subfamily. The Acrididae and Tettigonidae subfamilies are discriminated in the stacked columns.

A total of 35 species were recorded during this study: 33 species during transect sampling, and two additional species outside of the transects: *Aiolopus strepens* (Latreille, 1804) and *Pyrgomorpha conica* (Olivier, 1791). Two species are recorded for the first time for Portugal: *Dociostaurus hispanicus* Bolivar, 1898 and *Euryparyphes terrulentus* (Serville, 1838). Furthermore, *Platystolus martinezii* (Bolívar, 1873), first recorded around 100 years ago in Portugal (Aires and Menano 1916), is now recorded for the second time. The most representative orthopteran families are Acrididae, with 21 species belonging to six subfamilies and Tettigonidae with 10 species belonging to three subfamilies. The remaining families, Pamphagidae, Pyrgomorphidae, Gryllidae and Tetrigidae, are each represented by a single species (Figure 2). Furthermore, all six families are represented in tree plantations, while we have only found five families in fallows and four in habitat edges (see Suppl. material 1).

The number of species was broadly similar in fallows (29), edges (24) and tree plantations (27), despite the highest number of transects placed in fallows. Among the forest types, the most speciose were oak plantations (21), whereas only 13 species were found in eucalyptus plantations. The lowest value of Grasshopper Conservation Index (GCIn) was found for eucalyptus plantations (0.44±0.12) and pine plantations (0.44±0.12), while the highest was found for fallows (0.52±0.18) and mixed pine-oak plantations (0.49±0.21). An intermediate value of GCIn was found for edges (0.48±0.18) and oak plantations (0.47±0.14). The species with highest possible GCI value (1) was *Euryparyphes terrulentus*.

Twenty species were common to fallows, edges and plantations, while other species only occurred in a particular habitat. The species, *Aiolopus strepens*, *Oedaleus decorus* (Germar, 1826), *Platycleis affinis* Fieber, 1853, *Platystolus martinezii* and the Red-Listed species *Dociostaurus hispanicus* and *Platycleis falx* (Fabricius, 1775) were only recorded in fallows. The species *Truxalis nasuta* (Linnaeus, 1758), was only recorded in edges. Three species, *Platycleis intermedia* (Serville, 1838), *Pyrgomorpha conica* and *Tettigonia viridissima* (Linnaeus, 1758) were only recorded in tree plantations (see Suppl. material 1).

**Figure 3.**
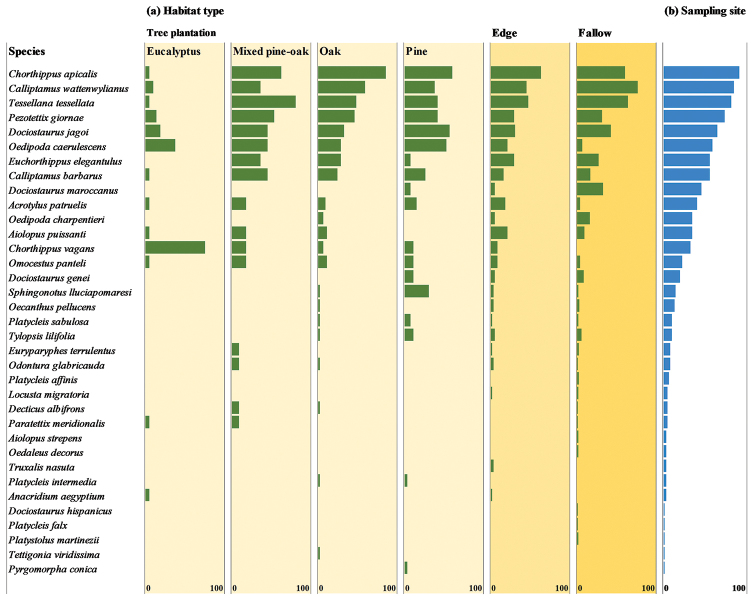
Frequency of occurrence (%) of each Orthoptera species per habitat type, calculated by dividing the number of occurrences by the number of transects in each habitat type (**a**), and percentage of sampling sites where the species occurred (**b**).

In terms of frequency of occurrence, the species Chorthippus (Glyptobothrus) apicalis (Herrich-Schaeffer, 1840), *Calliptamus wattenwylianus* Pantel, 1896 and *Tessellana tessellata* (Charpentier, 1825) were collected in the majority of the sampling sites (>80%) (Figure 3b). Overall, there was little variation in the frequencies of occurrence of these species among most of the habitat types, though there were generally lower frequencies in eucalyptus plantations. Within forest plantations, and specifically in eucalyptus plantations, *Chorthippus vagans* (Eversmann, 1848) occurred most frequently, possibly due to shade, patches of bare ground and sparse vegetation dominated by grasses characteristic of these plantations (Hochkirch et al. 2008). In pine plantations, Sphingonotus (Sphingonotus) lluciapomaresi (Defaut, 2005) was the species occurring most frequently. This is an Iberian endemic that occurs in areas with bare and rocky ground (Llucià-Pomares & Fernández-Ortín 2009), which characterize some of the pine plantations surveyed. In fallows, the species most frequently encountered was *Dociostaurus maroccanus* (Thunberg, 1815) (Figure 3a). This species typically occurs in dry grasslands, with patches of bare ground and short vegetation (Latchininsky 1998), which are created through livestock grazing. There are references from the 20^th^ century of *Dociostaurus maroccanus* as an agricultural pest, with population outbreaks recorded in Portugal and Spain (Silva 1947, Latchininsky 1998), and elsewhere in Europe (e.g., Nagy 1994). Although in recent decades *Dociostaurus maroccanus* has lost its economic importance, outbreaks may eventually occur again in the future under favourable ecological and agricultural management conditions (Latchininsky 1998, Aragón et al. 2013).

## Data resources

The data underpinning the analysis reported in this paper are deposited at GBIF, the Global Biodiversity Information Facility, http://ipt.gbif.pt/ipt/archive.do?r=orthopt_castroverde (doi: 10.15468/byd0kt, http://www.gbif.org/dataset/65f32597-48a7-4877-ae13-e4256b51fb6b).

## Material examined

### Family Tettigoniidae Krauss, 1902

#### Subfamily Tettigoniinae Kirby, 1906

***Decticus albifrons* (Fabricius, 1775)** IUCN category: Least Concern

**Material examined. A29**: 15/05/2015, OAK (1 M). **A79**: 26/05/2015, FAL (1 F). **P20**: 24/05/2014, MIX (1 M).

**Iberian distribution.** Widespread and very common all over the Peninsula.

***Platycleis affinis* Fieber, 1853** IUCN category: Least Concern

**Material examined. A68**: 20/06/2015, FAL (1 F). **B5**: 16/06/2015, FAL (1 F). **P1**: 25/06/2014, FAL (1 F). **P46**: 16/06/2015, FAL (1 F).

**Iberian distribution.** Present all over the Peninsula, except in the Pyrenees.

***Platycleis falx* (Fabricius, 1775)** IUCN category: Vulnerable B2ab(ii,iii,iv,v)

**Material examined. B8-1**: 21/06/2015, FAL (1 F).

**Iberian distribution.** Occurs mostly in the south-eastern part of the Iberian Peninsula and, specifically, it is known to occur in the central region of Portugal (Hochkirch et al. 2016). Our record expands this species’ distribution range to southern Portugal.

***Platycleis intermedia* (Serville, 1838)** IUCN category: Least Concern

**Material examined. A85**: 21/06/2015, OAK (1 F). **P52**: 31/05/2014, PIN (1 F).

**Iberian distribution.** This species occurs throughout continental Spain, except in the Northern region with a more Atlantic climate influence and most of the Pyrenees. Prior to this research, the species was only accounted for in the central region of Portugal. However, our study suggests that this species may have a larger distribution range in southern Portugal.

***Platycleis sabulosa* Azam, 1901** IUCN category: Least Concern

**Material examined. P15**: 18/06/2014, EDG (1 F). **P22**: 25/06/2014, PIN (1 F). **P23**: 18/06/2015, FAL (1 F). **P48**: 24/06/2014, OAK (1 F). **P51**: 24/06/2014, PIN (1 F). **P52**: 25/06/2014, FAL (1 F).

**Iberian distribution.** In Spain, it is widespread in the south and centre, but is apparently scarce in the northern regions. In Portugal, the species occurs from the centre to the south.

***Tessellana tessellata* (Charpentier, 1825)** IUCN category: Least Concern

**Material examined. A29**: 22/06/2015, FAL (2 F), OAK (1F). **A33**: 20/06/2015, FAL (1 F, 1M), PIN (1 F). **A57**: 20/06/2015, EDG (1 F), FAL (1F). **A64**: 22/06/2015, FAL (1 F, 2 M). **A68**: 20/06/2015 FAL (3 F), OAK (2 M). **A76**: 27/05/2015, FAL (3 M); 19/06/2015, EDG (1 M), FAL (2 F, 1 M). **A78**: 21/06/2015, FAL (1 F). **A79**: 23/06/2015, FAL (2 M), PIN (1 F). **A85**: 21/06/2015, FAL (2 F), EDG (1 F), OAK (1 F). **A90**: 24/06/2014, FAL (4 M), EDG (1 M). **A98**: 19/06/2015, FAL (2 F), EDG (1 M), PIN (1 M). **A99**: 19/06/2015, EDG (1 F), FAL (2 F). **A152**: 15/06/2015, EDG (1 M), FAL (1 F, 1 M), OAK (1 F, 2 M). **B1**: 16/06/2015, FAL (1 F). **B5**: 16/06/2015, FAL (2 F, 1 M). **B6**: 18/06/2015, FAL (1 F, 2 M). **B7**: 18/06/2015, FAL (2 F, 1 M). **B8-1**: 21/06/2015, FAL (1 F, 2 M). **B13-2**: 22/06/2015, FAL (1 F, 1 M), EDG (1 M), MIX (1 M). **B14**: 01/06/2015, FAL (1 F, 2 M); 23/06/2015, FAL (1 F, 1 M). **P1**: 03/06/2014, FAL (1 F, 3 M), EDG (2 M); 25/06/2014, FAL (2 F, 1 M), EDG (1 M). **P3**: 18/06/2015, FAL (3 F), EDG (1 F), OAK (1 F, 2 M). **P5**: 18/06/2014, FAL (1 F, 5 M). **P6**: 15/06/2014, FAL (1 F, 1 M), EDG (1 M), PIN (2 F, 2 M). **P8**: 16/06/2014, FAL (3 F, 5 M), EDG (3 F). **P9**: 20/06/2014, FAL (1 F, 5 M), EUC (1 F). **P11**: 20/06/2014, FAL (2 F, 4 M), EDG (1 F, 1 M), OAK (1 F, 1 M). **P12**: 20/06/2014, FAL (2 F, 2 M), EDG (1 M). **P13**: 16/06/2014, FAL (1 F, 3 M), EDG (2 M). **P15**: 18/06/2014, FAL (1 F, 1 M), EDG (1 F, 1 M), PIN (2 F). **P17**: 15/06/2014, FAL (2 F, 4 M), EDG (1 M). **P18**: 14/06/2014, FAL (4 F, 2 M), EDG (1 F, 2 M), OAK (4 F, 1 M). **P20**: 18/06/2014, FAL (3 F), EDG (1 F), MIX (1 F, 2 M). **P22**: 04/06/2014, FAL (2 F, 1 M), EDG (1 F), PIN (1 F, 1 M); 25/06/2014, FAL (1 F, 1 M), PIN (2 F). **P23**: 18/06/2015, FAL (1 M). **P24**: 14/06/2014, FAL (2 M). **P27**: 16/06/2015, FAL (2 F, 2 M). **P28**: 17/06/2014, FAL (6 F, 1 M), EDG (1 M), OAK (3 F, 1 M). **P29**: 19/06/2014, FAL (2 F). **P30**: 15/06/2015, FAL (1 F, 2 M), EDG (1 F), MIX (1 F). **P31**: 19/06/2014, FAL (1 M), MIX (2 F, 1 M). **P32**: 19/06/2014, FAL (3 F, 4 M), OAK (2 F, 2 M). **P36**: 24/06/2014, FAL (2 F). **P39**: 15/06/2015, FAL (2 F), EDG (1 F), OAK (1 M). **P46**: 16/06/2015, FAL (1 F, 1 M), EDG (1 M). **P47**: 16/06/2014 (3 F, 1 M), EDG (1 M), OAK (3 M). **P48**: 24/06/2014, FAL (2 F, 2 M), EDG, (1 M). **P49**: 01/07/2014, FAL (2 F, 1 M), EDG (1 F, 1 M), MIX (2 F, 1 M). **P51**: 24/06/2014, FAL (1 F, 5 M), EDG (1 F). **P52**: 25/06/2014, FAL (4 F), EDG (1 F, 1 M), PIN (1 F).

**Iberian distribution.** Widespread and very common all over the Peninsula.

***Tettigonia viridissima* (Linnaeus, 1758)** IUCN category: Least Concern

**Material examined. A85**: 12/05/2015, OAK (1 M).

**Iberian distribution.** Widespread and very common all over the Peninsula.

#### Subfamily Phaneropterinae Kirby, 1904

**Odontura (Odontura) glabricauda (Charpentier, 1825)** IUCN category: Least Concern

**Material examined. A63**: 26/05/2015, FAL (1 M); **B7**: 22/04/2015, FAL (1 F); **P18**: 17/04/2014, OAK (1 F); **P20**: 26/04/2014, MIX (1 M); **P39**: 16/04/2015, EDG (1 M).

**Iberian distribution.** This is an Iberian endemic species and is restricted to the southwestern quadrant of the Peninsula, where it is widespread and common.

***Tylopsis lilifolia* (Fabricius, 1793)** IUCN category: Least Concern

**Material examined. A29**: 22/06/2015, EDG (1 M), FAL (3 M), OAK (1 M); **A33**: 20/06/2015, EDG (2 M), FAL (3 F), PIN (1 M); **B7**: 18/06/2015, FAL (1 F, 1 M); **B8-1**: 21/06/2015, EDG (1 M); **P5**: 18/06/2014, FAL (1 F, 1 M); **P6**: 15/06/2014, PIN (2 M).

**Iberian distribution.** In Spain it occurs mostly in the southern Mediterranean regions; in Portugal it is mainly known from the central region. It was Seabra (1937, 1939) who cited this species from a locality called Évora, representing the southernmost record in Portugal. Thus, our findings expand this species’ distribution range to a southernmost locality in Portugal, which suggest a larger distribution range.

#### Subfamily Bradyporinae Burmeister, 1838

***Platystolus martinezii* (Bolívar, 1873)** IUCN category: Least Concern

**Material examined. A29**: 31/05/2015, FAL (1 F, 1 M).

**Iberian distribution.** This is an Iberian endemic species mainly distributed in the centre of the Peninsula (Barat 2012). Until now, the only record of this species in Portugal was provided by Aires and Menano (1916) in Portalegre, a central locality near the border with Spain. Therefore, our new record considerably expands its endemic distribution range to southern Portugal.

### Family Gryllidae Saussure, 1893

#### Subfamily Oecanthinae Kirby, 1906

***Oecanthus pellucens* (Scopoli, 1763)** IUCN category: Least Concern

**Material examined. B1**: 16/06/2015, FAL (1 F); **P3**: 18/06/2015, OAK (1 F); **P17**: 15/06/2014, FAL (1 F); **P29**: 19/06/2014, EDG (1 F, 1 M); **P30**: 15/06/2015, FAL (1 F); **P31**: 19/06/2014, FAL (1 F); **P51**: 24/06/2014, EDG (1 F); **P52**: 25/06/2014, FAL (1 F).

**Iberian distribution.** Widespread and very common all over the Peninsula.

### Family Tetrigidae Rambur, 1838

#### Subfamily Tetriginae Rambur, 1838

***Paratettix meridionalis* (Rambur, 1838)** IUCN category: Least Concern

**Material examined. B14**: 01/06/2015, EUC (2 F, 1 M); 23/06/2015, EUC (3 F, 2 M); **P31**: 19/06/2014, MIX (1 F, 2 M); **P52**: 25/06/2014, FAL (1 F).

**Iberian distribution.** Widespread and very common all over the Peninsula.

### Family Pamphagidae Burmeister, 1840

#### Subfamily Pamphaginae Burmeister, 1840

***Euryparyphes terrulentus* (Serville, 1838)** IUCN category: Least Concern

**Material examined. P3**: 25/05/2015, EDG (1 M); **P13**: 22/05/2014, FAL (1 F, 1 M); **P15**: 02/05/2014, FAL (1 M); **P20**: 24/05/2014, MIX (1 F).

**Iberian distribution.** This species is new to Portugal. *E. terrulentus* was previously considered being endemic to Spain, where it is common in the south. This is the first record of *E. terrulentus* and the first confirmed record of *Euryparyphes* genus for Portugal, expanding its endemic Iberian distribution to southern Portugal.

### Family Pyrgomorphidae Brunner, 1893

#### Subfamily Pyrgomorphinae Brunner von Wattenwyl, 1874

***Pyrgomorpha conica* (Olivier, 1791)** IUCN category: Least Concern

**Material examined. A76**: 05/05/2015, PIN (1 F).

**Iberian distribution.** Present in most of the Peninsula, but seems to be absent in a few regions of high altitude in the North.

### Family Acrididae Werner, 1936

#### Subfamily Acridinae Uvarov, 1926

***Truxalis nasuta* (Linnaeus, 1758)** IUCN category: Least Concern

**Material examined. A33**: 17/05/2015, EDG (1 M), 28/05/2015 (1 M); **P36**: 30/05/2014, EDG (1 F).

**Iberian distribution.** Widely distributed throughout the whole of Portugal, except for a few localities in the North. In Spain it has a meridional distribution, occurring in the north-eastern part of the country and in the southern half, although it seems to be absent in the centre.

#### Subfamily Calliptaminae Harz, 1975

***Calliptamus barbarus* (Costa, 1836)** IUCN category: Least Concern

**Material examined. A29**: 22/06/2015, OAK (3 F); **A33**: 20/06/2015, EDG (1 F), FAL (1 F, 2 M); **A64**: 22/06/2015, FAL (1 M); **A76**: 19/06/2015, EDG (1 F), FAL (1 F), PIN (1 M); **A78**: 21/06/2015, FAL (1 F); **A85**: 21/06/2015, FAL (3 F, 2 M), OAK (1 M); **A90**: 24/06/2014, FAL (1 F); **A98**: 19/06/2015, PIN (1 F, 2 M); **A99**: 19/06/2015, FAL (1 F); **A152**: 15/06/2015, FAL (1 F, 1 M); **B7**: 18/06/2015, EDG (2 M); **B8-1**: 21/06/2015, FAL (3 F, 1 M), EUC (1 M); **B13-2**: 22/06/2015, EDG (1 F), MIX (1 F); **B14**: 23/06/2015, FAL (1 F); **P5**: 18/06/2014, FAL (1 F); **P6**: 15/06/2014, PIN (3 M); **P11**: 20/06/2014, OAK (4 F, 4 M); **P13**: 16/06/2014, EDG (1 F); **P15**: 18/06/2014, PIN (2 M); **P18**: 14/06/2014, EDG (1 M), OAK (1 M); **P20**: 18/06/2014, EDG (1 M), MIX (1 F, 1 M); **P22**: 04/06/2014, EDG (1 M), 25/06/2014, FAL (2 M); **P23**: 18/06/2015, FAL (1 M); **P27**: 16/06/2015, FAL (1 F); **P28**: 17/06/2014, EDG (1 M), FAL (1 F); **P29**: 19/06/2014, FAL (1 M); **P30**: 15/06/2015, MIX (1 F); **P31**: 19/06/2014, FAL (3 M); **P32**: 19/06/2014, OAK (2 M); **P39**: 15/06/2015, OAK (1 F); **P46**: 16/06/2015, PIN (1 M); **P48**: 24/06/2014, EDG (1 F, 2 M), FAL (1 M), OAK (1 F, 1 M); **P49**: 01/07/2014, EDG (1 F), MIX (2 F, 1 M); **P52**: 25/06/2014, FAL (1 F, 4 M).

**Iberian distribution.** Widespread and very common all over the Peninsula.

***Calliptamus wattenwylianus* Pantel, 1896** IUCN category: Least Concern

**Material examined. A29**: 31/05/2015, EDG (1 M), FAL (1 F, 1 M), 22/06/2015, EDG (1 F), FAL (2 F, 1 M), OAK (2 F); **A33**: 28/05/2015, FAL (2 F, 1 M), 20/06/2015, FAL (4 M); **A57**: 20/06/2015, EDG (2 M), FAL (2 F, 1 M); **A64**: 01/06/2015, FAL (2 F, 3 M), 22/06/2015, FAL (1 F, 2 M), OAK (1 F); **A68**: 20/06/2015, FAL (2 M), OAK (1 M); **A76**: 27/05/2015, FAL (3 F, 6 M), EDG (1 M), PIN (2 M), 19/06/2015, FAL (2 M), EDG (1 M), PIN (1 M); **A78**: 30/05/2015, FAL (3 F, 3 M), PIN (1 F), 21/06/2015, FAL (1 F, 1 M), EDG (1 M), PIN (1 F); **A79**: 25/05/2015, EDG (1 M), 26/05/2015, FAL (3 F, 5 M), 23/06/2015, FAL (1 F, 2 M); **A85**: 30/05/2015, FAL (2 F, 3 M), EDG (1 F), OAK (1 F, 1 M), 21/06/2015, FAL (1 F, 2 M), EDG (1 M), OAK (1 M); **A90**: 24/06/2014, FAL (1 F), EDG (1 F); **A98**: 27/05/2015, FAL (4 F, 4 M), EDG (1 F, 1 M), PIN (3 M), 19/06/2015, FAL (2 M), EDG (1 M), PIN (1 M); **A99**: 25/05/2015, FAL (2 M), 19/06/2015, FAL (3 M), EDG (1 M); **A152**: 15/06/2015, FAL (1 F, 6 M), OAK, (1 F, 3 M); **B1**: 16/06/2015, FAL (4 F, 1 M), EDG (1 M); **B5**: 16/06/2015, FAL (4 M); **B6**: 22/05/2015, FAL (1 F, 2 M), 18/06/2015, FAL (1 F, 2 M), EDG (1 M), EUC (1 F); **B7**: 18/06/2015, EUC (2 M); **B8-1**: 16/05/2015, FAL (1 F), 30/05/2015, FAL (1 F, 4 M), 21/06/2015, FAL (2 F, 1 M); **B13-2**: 31/05/2015, FAL (5 M), EDG (2 M), 22/06/2015, FAL (1 F, 2 M), EDG (1 M), MIX (1 M); **B14**: 01/06/2015, FAL (2 F, 2 M), 23/06/2015, FAL (1 F, 1 M), EDG (1 M), EUC (1 M); **P1**: 03/06/2014, FAL (3 F), 25/06/2014, FAL (3 F, 1 M); **P3**: 25/05/2015, FAL (3 F, 6 M), OAK (1 F, 2 M), 18/06/2015, FAL (1 F, 4 M), EDG (1 M), OAK (2 F, 2 M); **P5**: 18/06/2014, FAL (4 F, 4 M); **P6**: 15/06/2014, FAL (6 F, 5 M); **P8**: 16/06/2014, FAL (1 F, 4 M), EDG (2 M), OAK (1 M); **P9**: 20/06/2014, FAL (2 F, 2 M); **P11**: 29/05/2014, FAL (4 F), EDG (1 M), OAK, (1 F, 2 M), 20/06/2014, FAL (2 M), EDG (1 M), OAK (2 F, 3 M); **P12**: 29/05/2014, FAL (1 F), 20/06/2014, FAL (2 F, 5 M), OAK (1 F); **P13**: 21/05/2014, PIN (1 F, 1 M), 22/05/2014, PIN (2 F, 1 M), 16/06/2014, FAL (1 F, 7 M), EDG (1 F, 3 M); **P15**: 25/05/2014, PIN (2 M), 26/05/2014, FAL (3 M), 18/06/2014, FAL (4 F, 3 M), EDG (1 M), PIN (1 F); **P17**: 15/06/2014, FAL (5 F, 4 M), EDG (1 M); **P18**: 14/06/2014, FAL (1 F, 8 M), OAK (5 F, 5 M); **P19**: 31/05/2014, FAL (2 F, 3 M), EDG (1 F, 1 M), OAK (1 F, 1 M); **P20**: 24/05/2014, MIX (1 F), 18/06/2014, FAL (4 F, 5 M), EDG (1 F, 1 M), MIX (2 F, 1 M); **P22**: 04/06/2014, FAL (6 F, 2 M), EDG (1 F), 25/06/2014, FAL (3 F, 7 M), EDG (1 M), PIN (2 M); **P23**: 22/05/2015, FAL (1 F, 3 M), 18/06/2015, FAL (1 F, 2 M), EDG (1 M); **P24**: 14/06/2014, FAL (1 M); **P27**: 16/06/2015, FAL (1 F, 1 M); **P28**: 17/06/2014, FAL (4 F, 4 M), EDG (1 M), OAK (2 F, 1 M); **P29**: 19/06/2014, FAL (2 F, 1 M); **P30**: 15/06/2015, FAL (2 F, 4 M); **P31**: 19/06/2014, FAL (5 F, 4 M), EDG (1 M), MIX (1 F); **P32**: 19/06/2014, FAL (6 F, 3 M), EDG (2 F), OAK (4 F, 4 M); **P36**: 30/05/2014, FAL (1 F), 24/06/2014, FAL (3 F, 4 M); **P39**: 15/06/2015, FAL (2 F), EDG (1 M), OAK (1 M); **P46**: 16/06/2015, FAL (1 F, 2 M); **P47**: 16/06/2014, FAL (5 F, 2 M), OAK (8 F, 4 M); **P48**: 24/06/2014, FAL (3 F, 1 M); **P49**: 01/07/2014, FAL (5 F, 3 M); **P50**: 01/06/2014, FAL (1 F); **P51**: 24/06/2014, FAL (3 F, 3 M); **P52**: 31/05/2014, FAL (3 F, 3 M), 25/06/2014, FAL (4 F, 7 M), EDG (1 F, 2 M), PIN (4 M).

**Iberian distribution.** Very abundant in the meridional half of the Peninsula but more scattered in the northern half, in the areas with more Atlantic influence.

#### Subfamily Pezotettiginae Uvarov, 1927

***Pezotettix giornae* (Rossi, 1794)** IUCN category: Least Concern

**Material examined. A29**: 22/06/2015, OAK (2 F); **A33**: 20/06/2015, FAL (3 F), EDG (1 F); **A57**: 20/06/2015, FAL (1 F); **A64**: 22/06/2015, FAL (1 F), EDG (1 F), OAK (1 F); **A68**: 20/06/2015, FAL (3 F), EDG (1 F), OAK (2 F); **A76**: 19/06/2015, PIN (1 F); **A78**: 30/05/2015, FAL (1 F), EDG (1 F), PIN (1 F); **A79**: 23/06/2015, PIN (1 F); **A85**: 30/05/2015, OAK (1 F), 21/06/2015, FAL (2 F), OAK (1 F); **A90**: 24/06/2014, FAL (1 F, 3 M); **A98**: 19/06/2015, PIN (1 F); **A99**: 19/06/2015, PIN (1 F); **A152**: 15/06/2015, FAL (4 F, 1 M), OAK (4 F); **B1**: 16/06/2015, FAL (1 F, 1 M); **B5**: 16/06/2015, FAL (2 F), EUC (1 M); **B6**: 18/06/2015, EDG (1 F); **B7**: 18/06/2015, EDG (1 F); **B8-1**: 30/05/2015, FAL (1 F, 1 M), 21/06/2015, FAL (1 F), EDG (1 F); **B13-2**: 22/06/2015, FAL (3 F), MIX (1 F); **B14**: 23/06/2015, FAL (1 F), EDG (1 M); **P1**: 25/06/2014, EDG (1 F); **P3**: 18/06/2015, FAL (1 M), OAK (1 F, 1 M); **P5**: 18/06/2014, FAL (1 F, 1 M), EUC (2 F, 1 M); **P6**: 15/06/2014, FAL (1 F), EDG (2 F), PIN (1 M); **P8**: 16/06/2014, FAL (1 F, 1 M); **P11**: 20/06/2014, FAL (1 F), EDG (1 F), OAK (1 F, 2 M); **P12**: 20/06/2014, FAL (3 F, 1 M), OAK (1 F, 1 M); **P15**: 18/06/2014, PIN (4 F); **P17**: 15/06/2014, FAL (1 F, 1 M); **P18**: 14/06/2014, FAL (4 F, 2 M), OAK (1 M); **P20**: 18/06/2014, EDG (2 F, 1 M), MIX (1 F); **P22**: 04/06/2014, FAL (1 F), PIN (1 F, 1 M), 25/06/2014, PIN (1 F); **P27**: 16/06/2015, EDG (1 F), EUC (1 F); **P28**: 17/06/2014, FAL (4 F), OAK (1 F); **P29**: 19/06/2014, FAL (1 F); **P30**: 15/06/2015, FAL (3 F), MIX (1 F); **P31**: 19/06/2014, FAL (2 F), MIX (2 F, 1 M); **P32**: 19/06/2014, FAL (2 F), EDG (1 F), OAK (7 F, 3 M); **P36**: 24/06/2014, FAL (1 F), EUC (1 M); **P39**: 15/06/2015, FAL (3 F), EDG (1 F), OAK (2 F); **P46**: 16/06/2015, FAL (2 F); **P47**: 22/04/2014, FAL (1 F), 16/06/2014, FAL (3 F), EDG (2 F); **P48**: 24/06/2014, EDG (1 F, 1 M); **P49**: 01/07/2014, FAL (2 F), MIX (1 F); **P51**: 24/06/2014, FAL (1 F), EDG (1 F).

**Iberian distribution.** This species is common and widely distributed throughout all of the Peninsula, particularly in the south.

#### Subfamily Cyrtacanthacridinae Harz, 1975

***Anacridium aegyptium* (Linnaeus, 1764)** IUCN category: Least Concern

**Material examined. B1**: 22/04/2015, EDG (1 F); **B5**: 20/04/2015, EUC (1 M).

**Iberian distribution.** Widespread and very common all over the Peninsula.

#### Subfamily Gomphocerinae Fieber, 1853

**Chorthippus (Glyptobothrus) apicalis (Herrich-Schaeffer, 1840)** IUCN category: Least Concern

**Material examined. A29**: 15/05/2015, FAL (3 F, 5 M), EDG (5 M), OAK (4 F, 4 M), 31/05/2015, FAL (1 F, 4 M), EDG (2 M), OAK (1 F, 2 M); **A33**: 17/05/2015, FAL (2 F, 5 M), EDG (1 F), PIN (3 M), 28/05/2015, FAL (2 M), EDG (1 M), PIN (1 M); **A57**: 05/05/2015, FAL (2 F, 3 M), 26/05/2015, FAL (2 M); **A63**: 14/05/2015, FAL (2 F, 5 M), EDG (1 M), 26/05/2015, FAL (1 F); **A64**: 14/05/2015, FAL (2 M), OAK (2 M); **A68**: 15/05/2015, EDG (2 M), OAK (2 F, 1 M), 28/05/2015, OAK (1 M); **A76**: 05/05/2015, FAL (2 F, 1 M), EDG (1 F, 2 M), PIN (1 F, 1 M); **A78**: 13/05/2015, FAL (2 F, 1 M), EDG (1 F, 4 M), PIN (1 F, 2 M); **A79**: 02/05/2015, FAL (3 F, 5 M), EDG (1 F, 1 M), PIN (2 F, 3 M); **A85**: 12/05/2015, FAL (1 F, 3 M), EDG (1 F, 5 M), OAK (1 F, 2 M); **A90**: 04/06/2014, FAL (1 M); **A98**: 13/05/2015, FAL (1 F, 2 M), EDG (2 M), PIN (1 F, 4 M); **A99**: 02/05/2015, FAL (2 F, 8 M), EDG (1 M); **A152**: 18/05/2015, FAL (2 M), EDG (1 M), OAK (2 M); **B1**: 24/05/2015, EDG (3 F); **B4**: 21/04/2015, FAL (1 M), EDG (1 M); **B5**: 20/04/2015, FAL (1 F, 1 M), 21/05/2015, FAL (1 F, 1 M), EDG (1 M); **B6**: 01/05/2015, FAL (2 F, 4 M), EDG (1 F), 22/05/2015, FAL (1 M); **B7**: 01/05/2015, FAL (2 F, 7 M), EDG (1 M); **B8-1**: 16/05/2015, FAL (1 F, 2 M); **B13-2**: 31/05/2015, EDG (1 F); **B14**: 01/06/2015, EDG (1 M); **P3**: 03/05/2015, FAL (2 F, 1 M), EDG (3 F, 1 M), OAK (12 F, 10 M), 25/05/2015, FAL (1 F, 1 M), OAK (2 F, 1 M); **P5**: 24/04/2014, FAL (1 F, 1 M); **P6**: 23/04/2014, FAL (1 M), PIN (1 F), 20/05/2014, PIN (1 F); **P8**: 22/04/2014, FAL (7 F, 7 M), 19/05/2014, FAL (2 F, 4 M), EDG (1 F, 3 M), OAK (1 F, 2 M); **P9**: 30/04/2014, FAL (1 F, 4 M), 23/05/2014, FAL (2 F, 1 M); **P10**: 30/04/2014, FAL (2 F, 6 M), EDG (1 F), OAK (2 F, 10 M), 17/05/2014, FAL (3 F, 4 M), EDG (2 M), OAK (1 F, 1 M); **P11**: 29/04/2014, FAL (5 F, 13 M), EDG (5 F, 5 M), OAK (2 F, 4 M), 29/05/2014, FAL (2 F), EDG (1 F); **P12**: 29/04/2014, FAL (3 F, 4 M), 29/05/2014, FAL (1 F, 1 M); **P13**: 25/04/2014, FAL (4 F, 1 M), EDG (1 F, 1 M), PIN (2 F, 4 M), 22/05/2014, FAL (2 F); **P15**: 02/05/2014, FAL (2 M), EDG (3 M), PIN (2 F, 3 M), 25/05/2014, PIN (1 F, 1 M); **P16**: 18/04/2014, FAL (1 M); **P17**: 19/04/2014, FAL (1 M), EDG (1 F), 16/05/2014, FAL (1 F, 1 M); **P18**: 17/04/2014, FAL (1 F, 2 M), EDG (2 F, 1 M), OAK (4 F, 3 M), 16/05/2014, FAL (3 M), EDG (1 F, 1 M), OAK (2 F, 5 M); **P19**: 09/05/2014, FAL (10 M), EDG (1 F, 2 M), OAK (9 M), 31/05/2014, OAK (1 M); **P20**: 25/04/2014, FAL (3 F, 6 M), EDG (1 F, 1 M), MIX (1 F), 26/04/2014, MIX (4 M), 24/05/2014, EDG (1 M); **P22**: 13/05/2014, FAL (1 F, 1 M), PIN (3 F, 6 M); **P23**: 18/04/2015, FAL (1 F, 4 M), EDG (1 M), 22/05/2015, FAL (2 F), EDG (2 F, 2 M); **P24**: 15/05/2014, FAL (1 F, 4 M); **P25**: 16/04/2015, FAL (1 F), OAK (1 M); **P28**: 22/05/2014, FAL (3 F, 1 M), EDG (1 M), OAK (1 M); **P29**: 27/05/2014, FAL (1 M); **P30**: 18/04/2015, MIX (1 F), EDG (1 M), 19/04/2015, FAL (2 F, 1 M), 21/05/2015, EDG (1 M); **P31**: 28/04/2014, MIX (1 M), 26/05/2014, EDG (2 M); **P32**: 01/05/2014, FAL (7 F, 6 M), EDG (1 M), OAK (10 F, 11 M), 27/05/2014, OAK (5 F, 3 M), 19/06/2014, OAK (1 M); **P33**: 26/04/2014, FAL (1 M), 17/05/2014, FAL (2 M), OAK (1 M); **P39**: 16/04/2015, FAL (2 M), OAK (2 M), 18/05/2015, FAL (2 F, 1 M), OAK (3 F, 2 M); **P42**: 14/05/2014, FAL (2 M); **P46**: 19/04/2015, FAL (2 M), EDG (1 F), PIN (1 M), 21/05/2015, FAL (2 F, 2 M); **P47**: 21/04/2014, OAK (1 F, 1 M), 19/05/2014, FAL (1 F, 1 M), OAK (2 F, 6 M); **P48**: 07/05/2014, FAL (1 M), EDG (3 F, 4 M), 08/05/2014, OAK (1 F, 1 M); **P49**: 08/05/2014, FAL (2 F, 1 M), EDG (1 F, 5 M), MIX (1 F, 6 M), 02/06/2014, EDG (1 F), MIX (3 F, 2 M); **P50**: 14/05/2014, EDG (1 M), OAK (1 F, 2 M); **P51**: 27/04/2014, FAL (1 F, 2 M), EDG (1 F); **P52**: 09/05/2014, FAL (1 F, 2 M), EDG (1 F, 1 M), PIN (4 M), 31/05/2014, FAL (1 F, 1 M).

**Iberian distribution.** Distributed in nearly all regions of the Peninsula, with the exception of the extreme north-west.

***Chorthippus vagans* (Eversmann, 1848)** IUCN category: Least Concern

**Material examined. A57**: 20/06/2015, EDG (2 M); **A63**: 14/05/2015, EUC (1 M), 26/05/2015, EUC (1 F); **A68**: 20/06/2015, OAK (1 F); **A74**: 03/06/2014, EUC (1 F, 2 M); **A90**: 24/06/2014, EUC (2 M); **B1**: 24/05/2015, EDG (1 M), 16/06/2015, EUC (3 F, 2 M); **B3**: 17/05/2015, PIN (1 M); **B5**: 21/05/2015, EUC (2 F), 16/06/2015, EUC (2 M); **B7**: 18/06/2015, EUC (2 F); **P5**: 23/05/2014, EUC (2 F, 1 M); **P6**: 15/06/2014, PIN (1 F, 1 M); **P9**: 22/05/2014, EUC (1 M), 20/06/2014 (1 F, 3 M); **P27**: 24/05/2015, EDG (1 F, 1 M), EUC (1 M), 16/06/2015, EDG (1 F, 1 M), EUC (1 F, 2 M); **P29**: 26/05/2014, EUC (1 F, 1 M), 19/06/2014, EDG (1 M), EUC (1 F, 2 M); **P30**: 15/06/2015, MIX (3 F); **P31**: 19/06/2014, MIX (1 F); **P36**: 30/05/2014, EUC (1 F, 1 M), 24/06/2014, EDG (1 F, 4 M), EUC (2 F, 1 M); **P39**: 15/06/2015, EUC (2 F, 1 M); **P42**: 14/05/2014, EUC (1 F); **P51**: 30/05/2014, PIN (1 F).

**Iberian distribution.** Widespread and very common all over the Peninsula.

***Dociostaurus genei* (Ocskay, 1833)** IUCN category: Least Concern

**Material examined. A78**: 21/06/2015, FAL (2 F, 1 M); **A79**: 23/06/2015, EDG (1 F, 1 M), FAL (3 M); **A98**: 19/06/2015, EDG (4 F, 1 M), FAL (8 F, 3 M); **A99**: 19/06/2015, FAL (4 F, 2 M); **A152**: 15/06/2015, FAL (1 M); **B8-1**: 21/06/2015, FAL (1 M); **B14**: 23/06/2015, FAL (1 F); **P13**: 16/06/2014, PIN (2 F); **P15**: 18/06/2014, FAL (6 F, 3 M); **P17**: 15/06/2014, PIN (1 F, 1 M); **P23**: 18/06/2015, FAL (1 F), PIN (2 F, 1 M); **P28**: 17/06/2014, EDG (1 M).

**Iberian distribution.** Present throughout most of the Peninsula with exception of the extreme north-west.

***Dociostaurus hispanicus* Bolivar, 1898** IUCN category: Near Threatened

**Material examined. P28**: 17/06/2014, FAL (1 F).

**Iberian distribution.** First record for Portugal. This is an Iberian endemic species, has in Spain has a more western distribution and can be found from north to south (García et al. 2005). Our record is the southernmost for the Iberian Peninsula, and suggests a wider distribution.

***Dociostaurus jagoi* Soltani, 1978** IUCN category: Least Concern

**Material examined. A29**: 22/06/2015, FAL (1 F), EDG (1 M), OAK (1 F); **A33**: 20/06/2015, FAL (3 F, 1 M); **A64**: 22/06/2015, FAL (2 F, 1 M), EDG (1 M); **A68**: 20/06/2015, FAL (1 F, 1 M), EDG (1 M); **A76**: 19/06/2015, FAL (4 F, 5 M), EDG (2 F), PIN (2 F); **A78**: 21/06/2015, FAL (2 F, 4 M), EDG (1 F, 2 M), PIN (2 F, 3 M); **A79**: 23/06/2015, FAL (5 F, 5 M), EDG (1 F, 1 M), PIN (1 F, 1 M); **A85**: 21/06/2015, FAL (5 F, 2 M), EDG (1 M); **A98**: 19/06/2015, FAL (3 F, 2 M), EDG (1 M), PIN (2 F, 2 M); **A99**: 19/06/2015, FAL (6 F, 3 M), EDG (1 F, 1 M); **A152**: 15/06/2015, FAL (3 M), OAK (1 M); **B6**: 18/06/2015, FAL (7 F, 16 M), EUC (1 F); **B7**: 18/06/2015, FAL (1 F), EUC (1 F); **B8-1**: 21/06/2015, FAL (3 F, 1 M), EUC (1 M); **B13-2**: 22/06/2015, FAL (2 F, 3 M), MIX (1 M); **B14**: 23/06/2015, FAL (2 F, 3 M); **P1**: 25/06/2014, FAL (5 F, 3 M); **P3**: 18/06/2015, FAL (3 M), EDG (1 M), OAK (1 F); **P5**: 18/06/2014, FAL (3 M), EDG (1 F, 1 M), EUC (1 M); **P6**: 15/06/2014, PIN (2 F, 2 M); **P9**: 20/06/2014, FAL (1 M); **P11**: 20/06/2014, FAL (4 F, 4 M), EDG (1 F, 1 M), OAK (7 F, 2 M); **P12**: 20/06/2014, FAL (4 F, 2 M), OAK (4 F, 5 M); **P13**: 16/06/2014, FAL (6 F, 1 M), EDG (1 M), PIN (1 M); **P15**: 18/06/2014, FAL (4 F), EDG (1 F, 1 M), PIN (2 F); **P18**: 14/06/2014, OAK (1 F, 2 M); **P20**: 18/06/2014, FAL (2 M), EDG (1 M), MIX (1 F, 2 M); **P22**: 25/06/2014, FAL (2 F, 4 M), PIN (2 M); **P23**: 18/06/2015, FAL (3 F, 5 M), EDG (1 F, 2 M), PIN (2 M); **P27**: 16/06/2015, EUC (3 M); **P28**: 17/06/2014, FAL (1 F, 1 M), OAK (1 F); **P31**: 19/06/2014, FAL (1 F), MIX (2 F, 1 M); **P32**: 19/06/2014, OAK (1 M); **P36**: 24/06/2014, FAL (1 F, 2 M), EDG (1 M); **P39**: 15/06/2015, FAL (12 F, 14 M), EDG (4 M), OAK (7 F, 7 M); **P46**: 16/06/2015, FAL (1 M), PIN (1 F); **P48**: 24/06/2014, FAL (1 F, 4 M), OAK (2 F, 3 M); **P49**: 01/07/2014, FAL (2 M), EDG (1 M), MIX (1 F, 2 M); **P51**: 24/06/2014, FAL (4 F, 3 M); **P52**: 25/06/2014, FAL (1 M).

**Iberian distribution.** The area of occurrence extends throughout the Peninsula, with the exception of the northern strip faced to the Atlantic Ocean.

***Dociostaurus maroccanus* (Thunberg, 1815)** IUCN category: Least Concern

**Material examined. A33**: 17/05/2015, FAL (1 M), 20/06/2015, FAL (1 M); **A76**: 27/05/2015, FAL (3 F, 4 M), PIN (1M), 19/06/2015, FAL (1 M), EDG (1 F); **A78**: 13/05/2015, FAL (2 F, 5 M), EDG (1 M), 30/05/2015, FAL (2 F, 2 M); **A79**: 26/05/2015, FAL (2 F, 3 M); **A85**: 30/05/2015, FAL (3 F, 1 M); **A98**: 13/05/2015, FAL (2 F, 1 M), 27/05/2015, FAL (3 F), EDG (1 F), PIN (1 F); **A99**: 25/05/2015, FAL (3 M), 19/06/2015, FAL (1 F, 2 M), **A152**: 15/06/2015, FAL (1 F); **B5**: 21/05/2015, FAL (1 F, 1 M), 16/06/2015, FAL (2 F, 1 M); **B6**: 22/05/2015, FAL (4 F, 1 M); **B13-2**: 31/05/2015, FAL (1 F, 1 M); **B14**: 01/06/2015, FAL (3 F, 1 M); **P3**: 25/05/2015, FAL (3 F, 4 M), 18/06/2015, FAL (2 M); **P15**: 26/05/2014, FAL (3 F, 3 M); **P17**: 16/05/2014, FAL (1 M), 15/06/2014, FAL (1 F); **P19**: 31/05/2014, FAL (2 F, 2 M); **P22**: 13/05/2014, FAL (3 F), 04/06/2014, FAL (1 F, 1 M); **P23**: 22/05/2015, FAL (3 F, 1 M), 18/06/2015, FAL (2 F, 1 M); **P24**: 14/06/2014, FAL (1 M); **P27**: 16/06/2015, FAL (1 M); **P28**: 22/05/2014, FAL (1 F), 17/06/2014, FAL (1 M); **P29**: 26/05/2014, FAL (1 F, 2 M), 19/06/2014, FAL (2 M); **P30**: 21/05/2015, FAL (1 F), 15/06/2015, FAL (1 M); **P39**: 18/05/2015, FAL (1 F), 15/06/2015, FAL (2 M); **P47**: 19/05/2014, FAL (1 M), 16/06/2014, FAL (2 F, 2 M); **P49**: 02/06/2014, FAL (1 F), 01/07/2014, FAL (1 F); **P50**: 01/06/2014, FAL (1 F, 2 M); **P52**: 31/05/2014, FAL (1 F).

**Iberian distribution.** Widely distributed in the Mediterranean area of the Peninsula up to north-eastern Portugal. Seems to be absent in the northern strip faced to the Atlantic Ocean of Spain.

***Euchorthippus elegantulus* Zeuner, 1940** IUCN category: Least Concern

**Material examined. A33**: 28/05/2015, FAL (1 F, 2 M), EDG (1 F), 20/06/2015, FAL (3 F, 1 M), EDG (1 F), PIN (2 M); **A57**: 20/06/2015, FAL (2 M); **A64**: 01/06/2015, FAL (1 M), 22/06/2015, FAL (2 F, 3 M), EDG (1 F, 2 M), OAK (1 M); **A68**: 20/06/2015, FAL (1 F, 5 M), EDG (1 M), OAK (2 M); **A85**: 21/06/2015, EDG (1 M); **A90**: 04/06/2014, FAL (2 M), 24/06/2014, FAL (2 F, 2 M); **A99**: 25/05/2015, FAL (1 M), EDG (1 F), 19/06/2015, FAL (3 F, 1 M); **A152**: 15/06/2015, FAL (5 F, 2 M), EDG (1 F, 4 M), OAK (1 F, 1 M); **B5**: 16/06/2015, FAL (1 F, 1 M); **B6**: 18/06/2015, FAL (1 F); **B8-1**: 30/05/2015, FAL (1 F); **B13-2**: 31/05/2015, FAL (1 F, 2 M), 22/06/2015, FAL (1 F, 3 M), EDG (1 F, 1 M); **B14**: 01/06/2015, EDG (1 M), 23/06/2015, FAL (1 M), EDG (2 M); **P3**: 18/06/2015, FAL (1 M); **P6**: 15/06/2014, EDG (1 M); **P9**: 20/06/2014, FAL (3 F, 2 M); **P11**: 29/05/2014, FAL (1 M), OAK (1 M); **P12**: 29/05/2014, FAL (1 M), 20/06/2014, FAL (1 F, 2 M); **P18**: 14/06/2014, EDG (1 M), OAK (4 F, 1 M); **P20**: 18/06/2014, EDG (1 F, 1 M), MIX (1 M); **P23**: 18/06/2015, FAL (3 M); **P27**: 16/06/2015, FAL (3 F, 4 M); **P28**: 17/06/2014, EDG (1 F, 1 M); **P29**: 19/06/2014, FAL (2 F, 1 M), EDG (1 M); **P30**: 15/06/2015, FAL (1 F, 2 M), EDG (1 F), MIX (1 F); **P31**: 19/06/2014, FAL (1 M), EDG (1 M), MIX (2 F, 3 M); **P32**: 19/06/2014, FAL (2 F, 3 M); **P36**: 30/05/2014, FAL (1 M), 24/06/2014, FAL (1 M); **P39**: 18/05/2015, EDG (1 M), 15/06/2015, EDG (2 F), OAK (3 F, 2 M); **P46**: 21/05/2015, PIN (1 M), 16/06/2015, FAL (3 F, 4 M), EDG (2 F, 3 M), PIN (3 M); **P47**: 16/06/2014, OAK (1 F, 2 M); **P48**: 24/06/2014, OAK (1 F); **P51**: 24/06/2014, FAL (2 F, 3 M); **P52**: 25/06/2014, FAL (1 F, 1 M), EDG (1 F, 1 M).

**Iberian distribution.** Present in almost in all of the Peninsula, being absent in the northern third.

***Omocestus panteli* (Bolivar, 1887)** IUCN category: Least Concern

**Material examined. A33**: 17/05/2015, FAL (2 F), PIN (1 F), 28/05/2015, EDG (1 F); **A64**: 14/05/2015, OAK (3 F, 4 M), 01/06/2015, OAK (3 M), 22/06/2015, OAK (1 M); **A90**: 04/06/2014, FAL (1 M); **A99**: 02/05/2015, EDG (2 M), PIN (1 M); **A152**: 18/05/2015, EDG (1 M); **B13-2**: 31/05/2015, FAL (2 M); **B14**: 01/06/2015, EDG (1 F, 1 M), EUC (1 M); **P25**: 16/04/2015, EDG (1 M); **P30**: 18/04/2015, MIX (1 F), 19/05/2015, FAL (1 F, 1 M); **P31**: 25/05/2014, MIX (1 M), 19/06/2014, MIX (1 F, 3 M); **P39**: 16/04/2015, OAK (2 F), 18/05/2015, OAK (6 F, 3 M); **P46**: 19/04/2015, PIN (1 F), 21/05/2015, FAL (1 M); **P50**: 01/06/2014, OAK (1 M); **P52**: 09/05/2014, FAL (1 M), 31/05/2014, FAL (2 F, 1 M).

**Iberian distribution.** This is an Iberian endemic species and occurs in most of the Iberian territory, with the exception of a few localities in the North, particularly in the Pyrenees.

#### Subfamily Oedipodinae

***Acrotylus patruelis* (Herrich-Schaffer, 1838)** IUCN category: Least Concern

**Material examined. A29**: 31/05/2015, EDG (1 F, 1 M), 22/06/2015, EDG (1 F, 1 M); **A33**: 28/05/2015,EDG (1 F, 2 M), PIN (1 M); **A79**: 25/05/2015, PIN (1 F), 26/05/2015, FAL (1 F); **A90**: 04/06/2014, EDG (1 F); **A99**: 25/05/2015, FAL (1 F); **A152**: 15/06/2015, EDG (1 F); **B1**: 24/05/2015, EDG (1 F), 16/06/2015, EDG (1 M); **B7**: 22/05/2015, EDG (2 F); **B8-1**: 16/05/2015, FAL (1 M); **B13-2**: 31/05/2015, FAL (1 M), 22/06/2015, FAL (1 F); **B14**: 01/06/2015, EUC (1 F); **P1**: 03/06/2014, FAL (2 F); **P3**: 25/05/2015, FAL (1 F); **P13**: 22/05/2014, PIN (1 F); **P19**: 31/05/2014, OAK (1 F), **P22**: 04/06/2014, PIN (1 F); **P23**: 22/05/2015, EDG (1 F); **P29**: 19/06/2014, FAL (1 F); **P30**: 21/05/2015, MIX (1 F); **P31**: 19/06/2014, MIX (1 F, 1 M); **P39**: 18/05/2015, EDG (1 F), OAK (2 F, 3 M), 15/06/2015, OAK (1 F); **P42**: 14/05/2014, EDG (1 M); **P50**: 01/06/2014, EDG (1 F, 1 M).

**Iberian distribution.** Distributed mostly in the meridional half and centre of Spain. In Portugal, it is apparently absent in the northern half of the country and in some localities of the littoral west of the South.

***Aiolopus puissanti* Defaut, 2005** IUCN category: Least Concern

**Material examined. A29**: 31/05/2015, EDG (1 F, 1 M); **A33**: 17/05/2015, FAL (2 F), EDG (2 M), 28/05/2015, EDG (1 M); **A57**: 20/06/2015, FAL (1 F, 1 M); **A64**: 01/06/2015, EDG (1 F); **A68**: 28/05/2015, FAL (2 F, 1 M); **A74**: 03/06/2014, FAL (1 F, 1 M), EDG (1 M); **B13-2**: 31/05/2015, FAL (2 F, 1 M), 22/06/2015, FAL (1 F); **B14**: 23/06/2015, EUC (1 F); **P1**: 03/06/2014, EDG (4 F, 2 M); **P10**: 17/05/2014, OAK (1 M); **P13**: 22/05/2014, FAL (1 M); **P23**: 22/05/2015, FAL (2 F, 2 M), EDG (1 M); **P29**: 26/05/2014, EDG (1 F, 1 M); **P30**: 19/05/2015, MIX (1 F), 21/05/2015, FAL (1 M), 15/06/2015, EDG (1 F); **P31**: 25/05/2014, MIX (1 M), 19/06/2014, MIX (1 F, 1 M); **P32**: 27/05/2014, EDG (1 F, 1 M); **P36**: 30/05/2014, EDG (1 M); **P39**: 16/04/2015, EDG (1 F), 18/05/2015, OAK (4 F); **P46**: 21/05/2015, FAL (3 F, 2 M), 16/06/2015, FAL (1 F); **P50**: 01/06/2014, EDG (1 F), OAK (2 F, 1 M); **P52**: 31/05/2014, FAL (1 F, 1 M), 25/06/2014, FAL (1 F).

**Iberian distribution.** Present in almost all of the Peninsula with exception of the northern third.

***Aiolopus strepens* (Latreille, 1804)** IUCN category: Least Concern

**Material examined. A74**: 03/06/2014, FAL (1 F); **B13-2**: 17/07/2015, FAL (1 F).

**Iberian distribution.** Widespread and very common all over the Peninsula.

***Locusta migratoria* (Fabricius, 1781)** IUCN category: Least Concern

**Material examined. A33**: 28/05/2015, EDG (1 M); **P3**: 25/05/2015, FAL (1 F), 18/06/2015, FAL (1 M); **P46**: 16/06/2015, FAL (1 F).

**Iberian distribution.** Widespread and very common all over the Peninsula.

***Oedaleus decorus* (Germar, 1826)** IUCN category: Least Concern

**Material examined. B13-2**: 22/06/2015, FAL (1 M); **P31**: 19/06/2014, FAL (1 M).

**Iberian distribution.** Widespread and very common all over the Peninsula.

***Oedipoda caerulescens* (Linnaeus, 1758)** IUCN category: Least Concern

**Material examined. A29**: 31/05/2015, OAK (1 F), 22/06/2015, OAK (1 M); **A33**: 28/05/2015, FAL (2 F, 3 M), EDG (2 F), PIN (1 M), 20/06/2015, FAL (3 F), EDG (1 F), PIN (1 M); **A57**: 20/06/2015, EUC (1 M); **A63**: 26/05/2015, EDG (3 M); **A68**: 20/06/2015, OAK (1 F); **A76**: 27/05/2015, PIN (1 M), 19/06/2015, PIN (1 F); **A78**: 13/05/2015, PIN (1 M), 30/05/2015, FAL (1 F), PIN (1 F), 21/06/2015, PIN (1 F, 2 M); **A79**: 25/05/2015, PIN (1 M), 26/05/2015, FAL (1 M), 23/06/2015, PIN (1 M); **A85**: 30/05/2015, OAK (2 F); **A90**: 24/06/2014, EDG (1 M); **A98**: 27/05/2015, PIN (1 M); **A99**: 25/05/2015, EDG (1 F), PIN (1 F), 19/06/2015, EDG (1 M), FAL (2 F); **A152**: 15/06/2015, FAL (1 F); **B1**: 16/06/2015, EDG (1 F), EUC (2 F, 2 M); **B5**: 16/06/2015, EUC (3 M); **B6**: 18/06/2015, EDG (1 M), EUC (1 F, 4 M); **B13-2**: 22/06/2015, MIX (1 M); **B14**: 01/06/2015, EUC (1 M), 23/06/2015, EUC (1 M); **P3**: 18/06/2015, EDG (1 M); **P5**: 18/06/2014, EDG (2 M); **P6**: 15/06/2014, PIN (1 F); **P9**: 20/06/2014, EUC (2 M); **P13**: 16/06/2014, PIN (1 F); **P17**: 15/06/2014, FAL (1 F), PIN (1 M); **P18**: 14/06/2014, OAK (4 F, 1 M); **P20**: 18/06/2014, MIX (1 F); **P22**: 04/06/2014, PIN (1 F, 1 M), 25/06/2014 (1 F, 1 M); **P24**: 13/06/2014, EDG (2 F); **P27**: 16/06/2015, EDG (1 F), EUC (1 F, 1 M); **P29**: 19/06/2014, EDG (1 F), EUC (1 M); **P30**: 15/06/2015, MIX (1 F, 2 M); **P32**: 19/06/2014, OAK (1 F, 1 M); **P39**: 18/05/2015, EDG (1 M), 15/06/2015, FAL (2 F, 2 M), OAK (7 F, 6 M); **P47**: 16/06/2014, FAL (1 F); **P48**: 02/06/2014, OAK (2 F, 1 M), 24/06/2014, OAK (2 F, 3 M); **P49**: 01/07/2014, MIX (1 F, 1 M).

**Iberian distribution.** Widespread and very common all over the Peninsula.

***Oedipoda charpentieri* Fieber, 1853** IUCN category: Least Concern

**Material examined. A76**: 27/05/2015, FAL (1 M), 19/06/2015, FAL (2 F, 1 M); **A78**: 30/05/2015, FAL (6 F); **A79**: 26/05/2015, FAL (4 M); **A85**: 21/06/2015, OAK (1 F); **A90**: 04/06/2014, EDG (1 M); **A98**: 27/05/2015, FAL (2 F, 3 M), 19/06/2015, FAL (3 F, 1 M); **A99**: 19/06/2015, FAL (3 F); **B6**: 18/06/2015, FAL (2 F, 3 M); **B14**: 01/06/2015, FAL (1 M); **P1**: 25/06/2014, FAL (1 F); **P6**: 15/06/2014, FAL (2 F, 2 M); **P11**: 29/05/2014, FAL (1 F), OAK (1 F, 1 M); **P15**: 18/06/2014, FAL (1 M); **P17**: 15/06/2014, FAL (1 F); **P19**: 31/05/2014, FAL (2 F); **P20**: 18/06/2014, EDG (1 F); **P22**: 25/06/2014, FAL (1 M); **P23**: 22/05/2015, FAL (1 F), 18/06/2015, FAL (2 M); **P32**: 19/06/2014, FAL (1 M); **P47**: 16/06/2014, FAL (1 F); **P48**: 02/06/2014, EDG (1 M), 24/06/2014, FAL (1 M).

**Iberian distribution.** This species is widespread throughout the Mediterranean region of the Peninsula but is apparently absent in most of the extreme north-west, in the northern Atlantic littoral and in the Pyrenees.

**Sphingonotus (Sphingonotus) lluciapomaresi (Defaut, 2005)** IUCN category: Least Concern

**Material examined. A76**: 19/06/2015, PIN (1 M); **A78**: 21/06/2015, EDG (1 M), PIN (2 F); **A79**: 23/06/2015, PIN (1 M); **A98**: 19/06/2015, FAL (1 M), PIN (1 M); **A99**: 19/06/2015, FAL (1 M); **P12**: 20/06/2014, OAK (1 M); **P13**: 16/06/2014, PIN (1 M); **P22**: 25/06/2014, PIN (1 F, 1 M); **P23**: 18/06/2015, EDG (2 F, 2 M), PIN (1 F).

**Iberian distribution.** This species is endemic to the Iberian Peninsula and has a wide and central distribution in the Peninsula, occurring also in the south of Spain. Our record is the south-westernmost, extending the species known distribution range.

## Discussion

The results of the present study expand the list of Orthoptera species known for Portugal, and the species richness recorded augment the relevance of Castro Verde SPA in terms of biodiversity. The 35 species recorded are distributed among six of the 11 families known to occur in Portugal. From the species recorded, two are Red-Listed as threatened or near-threatened at the European level, and five are Iberian endemics. The diversity of species observed was probably driven by landscape heterogeneity, as we found species exclusive of both fallow land and forest plantations. Overall, our results point out the importance of Castro Verde SPA for the conservation of Orthoptera, and help identify some threats that may affect this value in the near future.

While the present study highlights the value of Castro Verde SPA in terms of Orthoptera diversity, it is likely that other species are present in the area. A more comprehensive species list can be obtained by using other methods, given the distinct phenologic and ecologic characteristics of certain groups and their ability for crypsis. For instance, beating should be used to collect tree and shrub-dwelling species, which are often difficult to detect. The use of bioacoustic exploration can also be advantageous to obtain information on less conspicuous orthopterans such as crickets and bush-crickets (e.g. *Gryllotalpa* spp.). In fact, despite the occurrence of *Gryllotalpa* spp. in Castro Verde SPA, which is referred in the work of Catry et al. (2012) as a prey of the Lesser Kestrel, the species was not detected in this study. Moreover, carrying out surveys during a broader day time frame should also increase the number of species detected, as some orthopterans are generally more active during the crepuscular and nocturnal periods of the day (e.g. crickets and bush-crickets). Broadening the surveys to other months of the year would also increases the probability of recording different species. The mature forms of some species can be found in autumn and early spring, like *Thyreonotus bidens* (Bolívar, 1887), whose adults typically occur in autumn (September-November). This species is likely to occur in Castro Verde SPA, as it is usually found in forests with undergrowth (Llucià-Pomares and Fernández-Ortín 2009).

The number of orthopteran species recorded in the Castro Verde SPA appeared to be high, and is similar to results of a few other studies carried out in the South of Portugal: 37 species in Setúbal municipality (Cordeiro 1914); 40 species in Monchique (Ebner 1941) and 37 species in Parque Natural da Ria Formosa (Lock 1999). However, the richness recorded in Castro Verde was much lower than that observed in the Sabor watershed (NE Portugal), where 64 species were found, albeit over a period of about 10 years and in a much larger area encompassing a wider variety of habitats (Miranda-Arabolaza et al. 2005). Clearly, further studies using standardised techniques are required to assess how the species richness varies across regions and habitats in Portugal, and what are the current ecological and anthropogenic drivers of such variation, as little information is available.

Out of the 35 species recorded, two are considered threatened or near-threatened under the IUCN criteria (*Platycleis falx* and *Dociostaurus hispanicus*) and five are Iberian endemics: *Dociostaurus hispanicus*, *Euryparyphes terrulentus*, *Omocestus panteli*, *Platystolus martinezii* and *Sphingonotus lluciapomaresi*. The presence of these elements underpins the natural value of Castro Verde SPA, as endemic and threatened species are often used in marking biodiversity hotspots and prioritizing areas for conservation (Myers 2000). Some of our records complement the current knowledge on species geographic distribution in southern Europe. Five species occurring in southern Spain expanded their known distribution to southern Portugal, namely: *Euryparyphes terrulentus*, *Platycleis falx*, *Platycleis intermedia*, *Sphingonotus lluciapomaresi* and *Tylopsis lilifolia*. The two endemic species, *Platystolus martinezii* and *Dociostaurus hispanicus*, previously recorded mostly in the centre of the Iberian Peninsula, extend their known distribution to the south-westernmost location in Europe. These new data referring to species diversity and distribution reflect the scarcity of studies on the orthopterofauna in Portugal, and indicate that some species could have a wider distribution in the Iberian Peninsula than is currently known.

Our results have also shown great variation among habitats in terms of species richness and conservation value. The maximum Grasshopper Conservation Index (GCIn; 0.52) and the maximum number of species (29) were found in fallows, whereas the maximum GCIn (0.49) in plantations was found in mixed pine-oak forests. The importance of fallows is also highlighted by the presence of uncommon species, which includes the two species of conservation concern recorded in our study, *Dociostaurus hispanicus* and *Platycleis falx*. In contrast to fallows, mixed pine-oak plantations had a relatively low species richness (16), with the high GCIn observed therein being a consequence of the high number of endemic, rare and flightless species. For instance, within forest plantations the species with the highest GCIn, *Euryparyphes terrulentus*, was only found in mixed pine-oak. The lowest value of both GCIn (0.44) and species richness (13) was observed in eucalyptus plantations. This was probably because eucalyptus plantations were composed by old trees, with a more closed canopy than other forest types. Moreover, the understorey often had overgrown and dense bushes and few grassy areas, and these conditions are largely unfavourable for open-area species that dominated the Orthoptera assemblages in our study area. These results are in line with other studies showing low abundance of macro-arthropods in *Eucalyptus* plantations in the Mediterranean region (Zahn et al. 2009). Overall, our results suggest that landscape heterogeneity was one of the drivers of orthopteran diversity observed in the Castro Verde SPA, as it provided opportunities for the persistence of species with different habitat affinities. Forest plantations played a role in this habitat mosaic, as they held species such as *Pyrgomorpha conica* and *Platycleis intermedia* that were absent elsewhere. However, as afforestation is considered one of the major threats to Orthoptera associated with open-land habitats, it is fundamental to preserve large unfragmented open habitats with forest patches kept to a minimum (Hochkirch et al. 2016, Bieringer et al. 2013).

The diversity of Orthoptera in the Castro Verde SPA, and particularly that of endemic species and species of conservation concern, may be at risk from ongoing changes in agricultural land uses. The two major land use changes that could threaten Orthoptera are: (i) changes in the rotational farming system, with a shift from the production of dry cereals and extensive sheep grazing on fallows and pastures, to the specialized production of sheep and cattle; and (ii) the expansion of permanent crops, particularly olive groves, though these occur mostly in the periphery of Castro Verde SPA due to legal restrictions within the area (Ribeiro et al. 2014, 2016). Afforestation of open farmland was a problem in the 1990s due to European subsidies under the regulation 2080/92 (Reino et al. 2010), but this is no longer affecting the region to a significant extent. It is uncertain how the ongoing changes will affect Orthoptera in the region, but it is likely that the intensification of land uses and the loss and fragmentation of important habitats such as fallows will greatly affect species richness and the abundance of some endemic species (Bieringer & Zulka 2003, Rook et al. 2004). Therefore, further studies would be necessary to understand the impacts of land use changes in orthopterans, which are needed for improving the management of the Castro Verde SPA.

Overall, our study provided new information on the diversity and ecology of Orthoptera in southern Portugal, providing clues for the conservation management of this group in the Castro Verde SPA and elsewhere in the country. First, a stronger basis is needed to ascertain the conservation status of Orthoptera in Portugal, which should be based on a Red List assessment carried out at the country level. Although the European Red List already provides some important information to assess what species are the most threatened (Hochkirch et al. 2016), there may be strong variation in conservation status at different spatial scales, thus requiring more detailed, regional assessments. This would be important to raise awareness about the value of the Portuguese Orthoptera, and also to identify the actions most needed to conserve this diverse group of insects. Second, the management of the Castro Verde SPA should take consideration of other taxonomic groups besides birds, which until now have been the main focus of attention (e.g. Reino et al. 2010, Santana et al. 2014, and references therein). As we have shown in this study, Orthoptera are a relevant part of biodiversity in the Castro Verde SPA, and so they should also be targeted when formulating new regulations on land use changes or designing agri-environmental subsidy schemes (Ribeiro et al. 2014). Third, surveys are needed on the Orthoptera fauna of other areas included in the Natura 2000 network, as this may be essential to assess the relevance and understand the threats affecting the conservation of this this and other arthropod groups, both in Portugal and elsewhere (Hochkirch et al. 2016). This is critical to undertake management actions that benefit the entire biodiversity, rather than only a few charismatic vertebrate species (e.g., Santana et al. 2014). Finally, we suggest that more attention should be given to the conservation of Orthoptera and other arthropods when designing the environmental components of the Common Agricultural Policy (CAP). As shown in the Castro Verde SPA, a large number of endemic and threatened species may be associated to low-intensity farmland systems (Weking et al. 2016), and thus these need to be maintained to assure the conservation of European biodiversity. Efforts are thus needed in the future to combine the conservation and agricultural policies, with the broader goal of preserving the rich diversity of species associated with open farmland landscapes such as those of the Castro Verde SPA.

## Supplementary materials

Supplementary material 1 Table S1. Species frequency of occurrence per habitat Data type: species data This dataset is made available under the Open Database License (http://opendatacommons.org/licenses/odbl/1.0/). The Open Database License (ODbL) is a license agreement intended to allow users to freely share, modify, and use this Dataset while maintaining this same freedom for others, provided that the original source and author(s) are credited.Sílvia Pina, Sasha Vasconcelos, Luís Reino, Joana Santana, Pedro Beja, Juan S. Sánchez-Oliver, Inês Catry, Francisco Moreira, Sónia Ferreira
